# Fixed-target serial femtosecond crystallography using *in cellulo* grown microcrystals

**DOI:** 10.1107/S2052252521005297

**Published:** 2021-06-18

**Authors:** J. Mia Lahey-Rudolph, Robert Schönherr, Miriam Barthelmess, Pontus Fischer, Carolin Seuring, Armin Wagner, Alke Meents, Lars Redecke

**Affiliations:** a Institute of Biochemistry, University of Lübeck, Ratzeburger Allee 160, 23562 Lübeck, Germany; b Center for Free-Electron Laser Science (CFEL), Deutsches Elektronen Synchrotron (DESY), Notkestrasse 85, 22607 Hamburg, Germany; c Photon Science, Deutsches Elektronen Synchrotron (DESY), Notkestrasse 85, 22607 Hamburg, Germany; d The Hamburg Center for Ultrafast Imaging, 22671 Hamburg, Germany; e Diamond Light Source, Diamond House DH2-52, Chilton, Didcot OX11 0DE, United Kingdom

**Keywords:** fixed-target SFX, serial femtosecond crystallography, *in cellulo* crystallography, intracellular protein crystals, silicon chip, Roadrunner

## Abstract

The crystallization of recombinant proteins in living cells is an exciting new approach in structural biology. Recent success has highlighted the need for fast and efficient diffraction data collection, optimally directly exposing intact crystal-containing cells to the X-ray beam, thus protecting the *in cellulo* crystals from environmental challenges. Serial femtosecond crystallography (SFX) at free-electron lasers (XFELs) allows the collection of detectable diffraction even from tiny protein crystals, but requires very fast sample exchange to utilize each XFEL pulse. Here, an efficient approach is presented for high-resolution structure elucidation using serial femtosecond *in cellulo* diffraction of micometre-sized crystals of the protein HEX-1 from the fungus *Neurospora crassa* on a fixed target. Employing the fast and highly accurate Roadrunner II translation-stage system allowed efficient raster scanning of the pores of micro-patterned, single-crystalline silicon chips loaded with living, crystal-containing insect cells. Compared with liquid-jet and LCP injection systems, the increased hit rates of up to 30% and reduced background scattering enabled elucidation of the HEX-1 structure. Using diffraction data from only a single chip collected within 12 min at the Linac Coherent Light Source, a 1.8 Å resolution structure was obtained with significantly reduced sample consumption compared with previous SFX experiments using liquid-jet injection. This HEX-1 structure is almost superimposable with that previously determined using synchrotron radiation from single HEX-1 crystals grown by sitting-drop vapour diffusion, validating the approach. This study demonstrates that fixed-target SFX using micro-patterned silicon chips is ideally suited for efficient *in cellulo* diffraction data collection using living, crystal-containing cells, and offers huge potential for the straightforward structure elucidation of proteins that form intracellular crystals at both XFELs and synchrotron sources.

## Abstract 2

A rapid fixed-target method is described for serial femtosecond crystallography diffraction data collection using *in cellulo* grown protein crystals directly within living insect cells.

## 1. Introduction   

During the past two decades, macromolecular X-ray crystallography (MX) has been revolutionized by the establishment of high-brilliance radiation sources and novel developments in serial diffraction data-collection strategies (Standfuss & Spence, 2017 ▸; Yamamoto *et al.*, 2017 ▸; Yabashi & Tanaka, 2017 ▸; Spence, 2020 ▸). The extremely short femtosecond pulses generated by X-ray free-electron lasers (XFELs) outrun radiation-damage effects, which limit the usable dose at synchrotron sources, enabling almost damage-free diffraction data collection at room temperature following the ‘diffraction-before-destruction’ approach (Neutze *et al.*, 2000 ▸; Chapman *et al.*, 2011 ▸). Immediately after a single high-energy XFEL pulse hits the sample, it is destroyed, essentially requiring continuous sample replenishment at the interaction region matching the pulse-repetition rate of the XFEL. This major challenge in XFEL diffraction experiments has been overcome in recent years by advancements in crystal-delivery strategies (Martiel *et al.*, 2019 ▸). In particular, advanced injection methods include, for example, liquid jets (DePonte *et al.*, 2008 ▸, 2011 ▸) and their developments (Oberthuer *et al.*, 2017 ▸; Knoška *et al.*, 2020 ▸), lipidic cubic phase (LCP; Weierstall *et al.*, 2014 ▸) and aerosol injectors (Bogan *et al.*, 2008 ▸; Hantke *et al.*, 2014 ▸), as well as hybrid methods, for example crystal extractors (Kovácsová *et al.*, 2017 ▸; Sugahara *et al.*, 2015 ▸; Park *et al.*, 2019 ▸; Conrad *et al.*, 2015 ▸) and tape drives (Roessler *et al.*, 2013 ▸; Beyerlein *et al.*, 2017 ▸), goniometer-based scanning using fixed-target supports (Zarrine-Afsar *et al.*, 2012 ▸; Hunter *et al.*, 2015 ▸; Roedig *et al.*, 2015 ▸, 2016 ▸; Murray *et al.*, 2015 ▸; Mueller *et al.*, 2015 ▸; Mehrabi *et al.*, 2020 ▸; Schulz *et al.*, 2018 ▸), and many more. The sequential collection of single-pulse diffraction images from thousands of individual crystals in random orientations subsequently merged into a three-dimensional data set enables the structure elucidation of biological macromolecules (Chapman *et al.*, 2011 ▸; Boutet *et al.*, 2012 ▸; Ayyer *et al.*, 2016 ▸). Based on the ultrashort X-ray pulses that diffract at high resolution even from crystals with dimensions in the submicrometre size range, this serial femtosecond X-ray crystallography (SFX) approach provides new opportunities for structural biology (Johansson *et al.*, 2017 ▸), including time-resolved X-ray diffraction to visualize the molecular dynamics of macromolecules by pump–probe experiments (Kupitz *et al.*, 2014 ▸; Tenboer *et al.*, 2014 ▸; Shimada *et al.*, 2017 ▸; Nango *et al.*, 2016 ▸). Sequential crystal-delivery strategies have successfully been adapted for serial synchrotron data collection to reduce dose accumulation in a single crystal, advancing the application of serial synchrotron crystallography (SSX) on microcrystals even at room temperature (Gati *et al.*, 2014 ▸; Stellato *et al.*, 2014 ▸; Roedig *et al.*, 2016 ▸; Owen *et al.*, 2017 ▸; Weinert *et al.*, 2017 ▸; Schulz *et al.*, 2018 ▸; Mehrabi *et al.*, 2019 ▸).

As a consequence of the increased X-ray intensities and serial data-collection strategies, small crystals formed within living cells, denoted ‘*in cellulo* crystals’, also became suitable targets for X-ray crystallography (reviewed in Schönherr *et al.*, 2018 ▸). Depending on the specific demands of the target crystals, diverse diffraction data-collection strategies have been reported for the elucidation of structural information from recombinant proteins that form intracellular crystals. At state-of-the-art synchrotron sources, serial mesh-screening approaches using microcrystals randomly positioned and frozen in a loop (Gati *et al.*, 2014 ▸) or on a mesh mount (Coulibaly *et al.*, 2007 ▸; Boudes *et al.*, 2016 ▸) have been applied. In 2012, the capability of SFX to generate new bioinformation was initially confirmed by the elucidation of the native *Trypanosoma brucei* procathepsin B (TbCatB) structure at 2.1 Å resolution using *in cellulo* grown and extracted crystals (Redecke *et al.*, 2013 ▸), followed by a number of additional examples highlighting the synergistic combination of SFX and intracellular protein crystallization (Gallat *et al.*, 2014 ▸; Ginn *et al.*, 2015 ▸; Gati *et al.*, 2017 ▸; Nass *et al.*, 2020 ▸). Motivated by the extraordinary intrinsic stability of the crystals that were used as the first diffraction targets, such as insect virus polyhedra (Coulibaly *et al.*, 2007 ▸), *in cellulo* grown crystals were usually isolated and purified from the cells prior to diffraction data collection to enable liquid-jet delivery. Moreover, isolation should avoid diffuse background scattering from the surrounding cellular material, which has been suggested to limit the recordable diffraction from the microcrystals (Duszenko *et al.*, 2015 ▸).

For the delivery of isolated *in cellulo* grown crystals into the XFEL beam, liquid-jet injectors that utilize gas dynamic virtual nozzles (GDVNs) have consistently been used, and this is the most commonly used method for SFX crystal injection to date (DePonte *et al.*, 2008 ▸). GDVNs inject at rapid flow rates (Schlichting, 2015 ▸), which is a benefit if fast reactions are being probed (Calvey *et al.*, 2016 ▸) or if ultrafast repetition rates need to be matched, for example at the European XFEL (Wiedorn, Awel *et al.*, 2018 ▸; Wiedorn, Oberthür *et al.*, 2018 ▸). On the other hand, rapid flow causes high sample consumption, as large quantities of crystals pass unexposed between subsequent X-ray pulses, significantly affecting the proportion of crystals hit by XFEL pulses. Typically, hundreds of thousands of small crystals are required to completely sample reciprocal space if the hit rate is less than 10%, as observed during GDVN injection of isolated *in cellulo* grown TbCatB (7.4%) and *T. brucei* IMP dehydrogenase (TbIMPDH; 2.3%) crystals (Redecke *et al.*, 2013 ▸; Nass *et al.*, 2020 ▸), which limits application to more challenging targets. Moreover, GDVN injectors tend to produce unstable jets due to clogging of the nozzle with crystals, and the aqueous environment usually results in an increased X-ray background scattering, which varies from frame to frame (Martiel *et al.*, 2019 ▸).

Aside from complications from the injector setup itself, the integrity of the protein samples can be affected by small environmental changes, as also observed for *in cellulo* crystals. Firefly luciferase and Xpa crystals grown in insect and mammalian cells, respectively, dissolve quickly after disruption of the cellular membrane (Schönherr *et al.*, 2015 ▸; Tsutsui *et al.*, 2015 ▸). On the other hand, high-quality diffraction data can be collected from *in cellulo* grown crystals that remain within the living cells without any impact from the cellular environment, as recently confirmed using synchrotron radiation (Tsutsui *et al.*, 2015 ▸; Baskaran *et al.*, 2015 ▸) and XFEL pulses (Sawaya *et al.*, 2014 ▸). Thus, it appears to be beneficial to exploit the protective effects of the viable cells on the intracellular crystals for diffraction data collection, instead of performing detrimental isolation and purification procedures. However, liquid-jet injection of intact crystal-containing cells at an XFEL is challenged by the comparatively large cell diameter (between 15 and 20 µm for insect cells), provoking increased settling and aggregation that might lead to rapid clogging of the jet nozzle.

In this study, we present a method for *in cellulo* SFX diffraction data collection that exploits the benefits of fixed-target sample delivery. To establish this approach, the selected test protein needs to fulfil specific requirements. Since the expected hit rate in the fixed-target SFX (FT-SFX) experiment directly correlates with the number of crystal-containing cells, crystals within every cell of the culture would be optimal. Only one crystal per cell would reduce the chance of multiple patterns per detector frame. Therefore, the protein should form intra­cellular crystals with dimensions in the low-micrometre size range in living cells reproducibly and with high efficiency to allow estimation of the crystallization efficiency by light microscopy. Finally, a reference structure should be available to validate the *in cellulo* FT-SFX approach. Considering these requirements, we have chosen the protein HEX-1 from the filamentous fungus *Neurospora crassa*, which represents a prominent example of native *in cellulo* protein crystallization (Schönherr *et al.*, 2018 ▸), as the crystallization target, employing a baculovirus-based approach for gene expression in insect cells.

In ascomycetes, HEX-1 self-assembles into a hexagonal crystalline core after import into peroxisome-derived compartments, named Woronin bodies, located in the vicinity of the septal pore (Markham & Collinge, 1987 ▸). The fungi exploit the stiffness and mechanical stability of the crystalline lattice to withstand the high intracellular turgor pressure to seal pores after damage to the organism, preventing cytoplasmic bleeding (Tenney *et al.*, 2000 ▸; Yuan *et al.*, 2003 ▸). Elucidation of the structure of HEX-1 recombinantly produced in in *Escherichia coli* and crystallized applying the sitting-drop vapour-diffusion method provided insights into the native assembly process (Yuan *et al.*, 2003 ▸). This HEX-1 structure determined from synchrotron diffraction of single crystals using the rotation method at cryogenic temperatures is subsequently denoted the HEX-1 reference structure. We recently reported that spontaneous self-assembly of HEX-1 into intracellular crystals is not restricted to the native environment of the fungal cells. On infection with a recombinant baculovirus encoding the HEX-1 gene, living insect cells also form regular, micrometre-sized hexagonal crystals (Lahey-Rudolph *et al.*, 2020 ▸), supporting a strong intrinsic crystallization tendency of HEX-1 that might have evolved along with its physiological function.

Sample supports for fast, reliable and efficient crystal delivery in FT-SFX and SSX have recently been described, particularly with regard to their increased hit rates and reduced background scattering compared with liquid-jet and LCP injection systems (Meents *et al.*, 2017 ▸; Lieske *et al.*, 2019 ▸; Tolstikova *et al.*, 2019 ▸; Mehrabi *et al.*, 2020 ▸). Diverse solid supports have already been designed based on silicon or polymers, including microgrids (Zarrine-Afsar *et al.*, 2012 ▸; Feld *et al.*, 2015 ▸; Baxter *et al.*, 2016 ▸) and silicon chips (Hunter *et al.*, 2015 ▸; Murray *et al.*, 2015 ▸; Roedig *et al.*, 2015 ▸, 2016 ▸, 2017 ▸; Schulz *et al.*, 2018 ▸; Karpik *et al.*, 2020 ▸; Shelby *et al.*, 2020 ▸; Frank *et al.*, 2014 ▸; Mehrabi *et al.*, 2020 ▸), and have successfully been established for isolated, conventionally grown protein crystals. We performed FT-SFX experiments at the Macromolecular Femtosecond Crystallography (MFX) end station of the Linac Coherent Light Source (LCLS), SLAC National Accelerator Laboratory, Menlo Park, California, USA (Sierra *et al.*, 2019 ▸) using crystal-containing insect cells loaded into the pores of micro-patterned, single-crystalline silicon chips in combination with the fast and accurate Roadrunner II translation-stage system (Lieske *et al.*, 2019 ▸). The location of the HEX-1 crystal-containing cells at predefined positions on the chip allowed fast and efficient raster scanning through the X-ray pulses, which were spatially aligned with the micro-pores of the chip by synchronization with the 120 Hz pulse-repetition rate of the LCLS (Roedig *et al.*, 2017 ▸). High hit rates of up to 30% (detector frames containing diffraction) resulted in short data-collection times and significantly reduced the sample consumption and the background scattering caused by non­crystalline material around the crystal compared with liquid-jet SFX. The overall agreement of the elucidated HEX-1 structure with that previously determined using synchrotron radiation and single HEX-1 crystals grown by sitting-drop vapour diffusion (PDB entry 1khi; Yuan *et al.*, 2003 ▸) validated our approach and demonstrated that FT-SFX is ideally suited for efficient *in cellulo* diffraction data collection using living, crystal-containing cells.

## 2. Materials and methods   

### 2.1. Insect-cell expression of *N. crassa* HEX-1 and *in cellulo* crystallization   

The cloning procedure and recombinant bacmid generation for HEX-1 from the filamentous fungus *N. crassa* (GenBank accession No. XM_958614) have previously been described (Lahey-Rudolph *et al.*, 2020 ▸). In brief, the HEX-1 coding sequence was amplified using primers 5′-TACTACGACGACGACGCTCACG-3′ (sense) and 5′-GAGGCGGGAACCGTGGACG-3’ (antisense) and blunt-end ligated into an EheI-restricted pFastBac1 vector containing the sequence 5′-ATGGGCGCCTAA-3′ between the BamHI and HindIII restriction sites. For recombinant baculovirus (rBV) production, recombinant bacmid DNA was generated in *E. coli* DH10EmBacY cells (Geneva Biotech) and used for lipofection of *Spodoptera frugiperda* Sf9 insect cells with Escort IV reagent (Sigma–Aldrich) according to the manufacturer’s instructions. The virus titre of the third-passage (P3) stock was calculated using the TCID_50_ (tissue-culture infectious dose; Reed & Muench, 1938 ▸) in a serial dilution assay as described previously (Lahey-Rudolph *et al.*, 2020 ▸).

**Figure 1:**
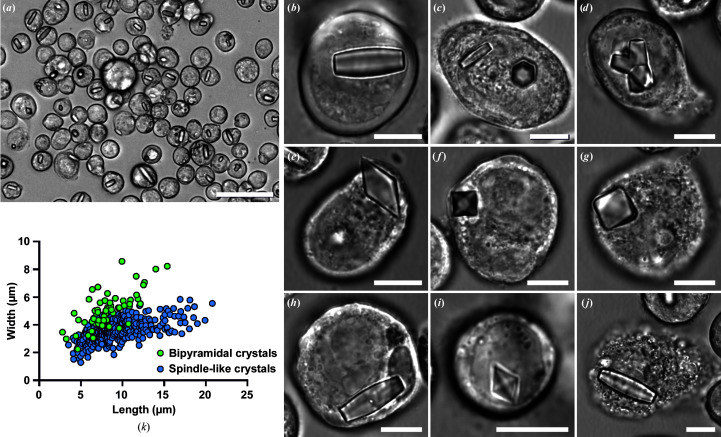
Crystallization of HEX-1 from *N. crassa* in living insect cells. Light-microscopic images of Sf9 cells four days after infection with a recombinant baculovirus encoding the target protein (MOI = 1). Most cells produce a single crystal per cell (*a*). Two different crystal morphologies can appear. The first is a spindle-like morphology with flat ends and a slightly increased diameter in the middle (*b*) showing a hexagonal cross section (*c*). These crystals sometimes grow in a star-like manner, conjoined in the middle (*d*). The second morphology is bipyramidal (*e*) with pointed tips and a square base (*f*). These crystals can sometimes form somewhat irregular forms with rounded tips (*g*). Both morphologies can grow cytoplasmatically as well as within the cell nucleus [(*b*) versus (*h*) and (*e*) versus (*i*)]. After cell lysis the crystals show high stability in the cell-culture medium, while mostly sticking to cell remnants (*j*). Bipyramidal crystals constitute about 13% of the total crystal population. The size distribution shows that the bipyramidal crystals form shorter but broader crystals overall compared with the spindle-like crystals (*k*). Size bars: 50 µm (*a*), 10 µm (*b*)–(*j*).

For intracellular crystallization, 0.8 × 10^6^ Sf9 insect cells were plated in 2 ml serum- and protein-free ESF921 insect-cell culture medium (Expression Systems) per six-well cell-culture plate and subsequently infected with recombinant P3 baculo­virus as a vector for HEX-1 with a multiplicity of infection (MOI) of 1. The cells were incubated at 27°C for 96 h. *In cellulo* crystal formation was verified and crystallization efficiency was determined by light-microscopic cell imaging using a Leica DM IL LED microscope equipped with a 40× objective employing integrated modulation contrast (Fig. 1 ▸).

### 2.2. Chip loading   

**Figure 2:**
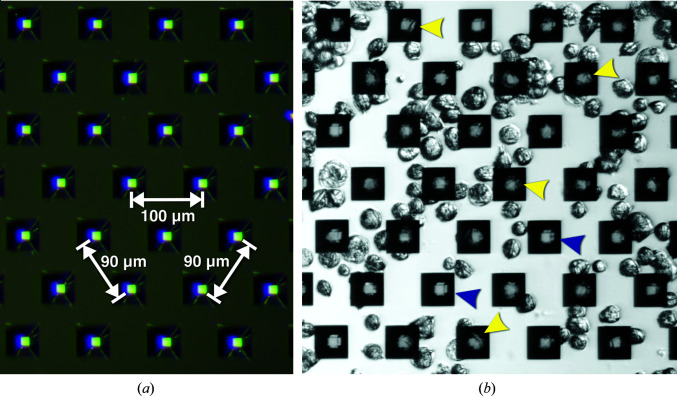
(*a*) An empty micro-patterned silicon chip designed for the Roadrunner II goniometer. The chip contains 12 µm sized pores that are spaced 90 and 100 µm apart, allowing well defined positioning of crystal-containing cells. (*b*) Sf9 insect cells containing *in cellulo* grown HEX-1 crystals were sucked into the chip pores. Visible intracellular crystals positioned in the pores are marked with yellow arrows; blue arrows point into empty pores. The images were recorded using reflected light on an Olympus SZX16 microscope.

For diffraction experiments, single-crystalline silicon microchips designed for the Roadrunner II goniometer (Tolstikova *et al.*, 2019 ▸) were used. Each 36 × 13 mm frame comprised a 300 µm thick chip with three 10 × 10 mm silicon windows thinned to 25 µm. These windows were patterned with a triangular grid of 12 µm pores spaced 90 and 100 µm apart, yielding approximately 44 000 pores that could hold crystal samples (Fig. 2 ▸). Prior to loading, the chips were glued onto an aluminium support.

Sf9 cells containing intracellular HEX-1 crystals were infected and incubated as described above before being transported to the MFX end station at room temperature. For loading onto one micro-patterned silicon chip, the cells were rinsed gently from the bottom of each well with a 1 ml pipette. After combining cells from three wells of a six-well culture plate into a reaction vessel, the cells were centrifuged for 1 min at 300*g*. The supernatant was discarded, and the cell pellet was gently resuspended in 200 µl ESF921 cell-culture medium and pipetted onto the chip surface. Utilizing a wedge cut from Whatman filter paper (grade 1), the medium was blotted off the back of the chip, pulling the cells into the 12 µm pores of the silicon support membrane. The cell coverage of the pores was confirmed by optical light microscopy of the chip using an Olympus SZX16 microscope illuminated by an Olympus KL 1600 LED (Fig. 2 ▸). Until just before loading the sample, the two cell-loaded chips were protected from dehydration using a ‘humidor’, a small movable cover that provides a humid environment (Lieske *et al.*, 2019 ▸).

### 2.3. FT-SFX data collection   

FT-SFX experiments were carried out at the MFX end station at LCLS (experiment No. P09215). X-ray pulses with a photon energy of 9.45 keV and a length of 27.5 fs were focused to a 1 × 1 µm spot. The pulse energy was attenuated to 20% of the full flux (4.5 mJ per pulse). Chips loaded with the crystal-containing cells were mounted on the high-precision Roadrunner II fast-scanning goniometer (Tolstikova *et al.*, 2019 ▸) in a humidified helium atmosphere to prevent dehydration of the cells during diffraction data collection. After manual alignment, data were collected at room temperature with a Cornell–SLAC pixel-array detector (CSPAD; Blaj *et al.*, 2015 ▸) by raster-scanning the chips through the X-ray focus row by row. The scanning speed was precisely controlled to synchronize the arrival of each X-ray pulse at the 120 Hz repetition rate of the LCLS with the spatial alignment of a micro-pore on the chip (Roedig *et al.*, 2017 ▸; Lieske *et al.*, 2019 ▸). By enclosing the direct beam shortly before and after the sample in thin-walled tantalum capillaries and by continuously flushing the Roadrunner humidity chamber with humidified helium gas, air scattering was further reduced to a minimum (Meents *et al.*, 2017 ▸). The sample-to-detector distance was set to 116 mm, corresponding to a maximum resolution of 2.0 Å at the detector edge. Higher resolution diffraction data were collected in the detector corners. Data collection was monitored using *OnDa* for immediate feedback in terms of the hit rate (Mariani *et al.*, 2016 ▸).

### 2.4. Data processing and structure refinement   

Identification of hits, indexing, integration and data reduction were performed using *indexamajig* as implemented in *CrystFEL* version 0.6.3 (White *et al.*, 2016 ▸), involving the application of the fast Fourier transform-based autoindexing algorithms *MOSFLM* (Leslie, 2006 ▸), *DirAx* (Duisenberg, 1992 ▸) and *XDS* (Kabsch, 2010 ▸). Concentric rings to determine background, buffer and peak estimation regions were set to 6.4, 5 and 3.4, respectively. To improve the data quality, the geometry of the CSPAD was refined using *geoptimiser* (Yefanov *et al.*, 2015 ▸). Frames containing more than three diffraction patterns of individual crystals were sorted out. The resulting stream file was subjected to post-refinement (scaling) and merged using *partialator* version 0.6.3, excluding saturated peaks above 14 300 ADU (analogue-to-digital units). Figures of merit were calculated using *compare_hkl* (*R*_split_, CC_1/2_ and CC*) and *check_hkl* (signal-to-noise ratio, multiplicity and completeness) from the *CrystFEL* suite (White *et al.*, 2016 ▸). Merged intensities obtained by *CrystFEL* were converted to MTZ format for further processing. Molecular replacement was performed with *Phaser* (McCoy *et al.*, 2007 ▸) using the HEX-1 structure previously obtained from crystals formed by conventional vapour diffusion as a search model (PDB entry 1khi; Yuan *et al.*, 2003 ▸), followed by iterative circles of refinement using *phenix.refine* (Afonine *et al.*, 2012 ▸) and manual model building with *Coot* (Emsley *et al.*, 2010 ▸). *Polygon* (Urzhumtseva *et al.*, 2009 ▸) and *MolProbity* (Chen *et al.*, 2010 ▸) were used for final model validation. The HEX-1 coordinates have been deposited in the PDB (accession codes 7asx and 7asi). Structural C^α^-atom overlays of residues 31–171 with the equivalent range of PDB entry 1khi were performed using *SUPERPOSE* from the *CCP*4 suite (Winn *et al.*, 2011 ▸) and were visualized in *PyMOL* (Schrödinger).

## 3. Results and discussion   

### 3.1. Intracellular crystallization of HEX-1   

In this study, our established *in cellulo* crystallization protocol was applied to produce quantities of intracellular HEX-1 crystals sufficient to elucidate its high-resolution structure by FT-SFX *in cellulo* diffraction. At day four post infection (p.i.) with the HEX-1 rBV, light-microscopy investigation of the Sf9 insect cells showed that 85% of the cells used for diffraction contained HEX-1 crystals, with a minor percentage of these cells harbouring multiple crystals [Fig. 1 ▸(*a*)]. Two crystal morphologies can be observed in infected insect cells: an elongated, spindle-shaped morphology with a hexagonal cross section [Figs. 1 ▸(*b*) and 1 ▸(*c*)] and a less often appearing bipyramidal shape with a square base [Figs. 1 ▸(*e*) and 1 ▸(*f*)]. Both formed in the cytoplasm or the nucleus [Figs. 1 ▸(*b*), 1 ▸(*e*), 1 ▸(*h*) and 1 ▸(*i*)]. The crystals exhibited average dimensions of 9.1 ± 3.2 µm in length and 3.5 ± 0.7 µm in width in the case of the spindle-like shape and 8.3 ± 2.5 µm in length and 5.1 ± 1.3 µm in width in the case of the bipyramidal crystals [Fig. 1 ▸(*k*)], not exceeding the normal dimensions of infected Sf9 cells (17.8 ± 2.6 µm) or affecting cell viability. In rare cases, spindle-shaped crystals grew up to a maximum length of 20 µm and a maximum width of 5.8 µm [Fig. 1 ▸(*k*)]. If cell lysis occurred due to the ongoing viral replication process, individual HEX-1 crystals floated in the medium or stayed attached to cell remnants, indicating significant crystal stability in the cell-culture medium [Fig. 1 ▸(*j*)]. However, free-floating HEX-1 crystals were rarely detected, in contrast to previous observations during the crystallization of TbIMPDH and TbCatB in living insect cells (Nass *et al.*, 2020 ▸; Redecke *et al.*, 2013 ▸), which might be attributed to the used bacmid. The TbIMPDH and TbCatB rBVs were generated applying the Bac-to-Bac system, while the HEX-1 gene was transposed into the EmBacY bacmid (Trowitzsch *et al.*, 2010 ▸). Due to the deletion of the viral cathepsin and chitinase genes, the generated baculovirus caused a reduced degree of cell lysis four days p.i.. The high intracellular crystallization efficiency and the comparatively narrow size range of the obtained *in cellulo* crystals, as well as the available reference structure, indeed qualify *N. crassa* HEX-1 as a suitable test protein to adapt FT-SFX to *in cellulo* diffraction data collection. In future studies, this intracellular diffraction data-collection approach may enable the structure solution of more delicate systems that lack the high intrinsic stability of HEX-1 crystals outside the protective cellular environment.

### 3.2. Sample loading onto micro-patterned silicon chips   

In terms of sample loading, crystal-containing insect cells behaved like isolated protein crystals, except for the increased dimensions of the cells (15–20 µm) that need to be considered during chip design. We used silicon chips designed for the Roadrunner II fast-scanning goniometer (Lieske *et al.*, 2019 ▸; Tolstikova *et al.*, 2019 ▸) with micro-pores of 12 µm in diameter to harbour the crystal-containing cells for pre-positioning. A spacing of 90 µm between the pores optimized the cell density to a single cell per pore on average. Since the surrounding liquor was efficiently removed by applying capillary forces from below by touching the bottom chip surface with a filter paper, the living insect cells were directly loaded onto the chip surface by carefully pipetting the cells suspended in culture medium. This loading procedure made washing steps or buffer exchange to fit the specific requirements of the diffraction experiment obsolete. Combining three wells of a six-well plate, each containing approximately 0.8 × 10^6^ Sf9 cells, resulted in the loading of 2.4 × 10^6^ cells per chip. This cell density optimized the cell coverage of the 44 000 pores of the chip after removal of the medium, as revealed by reflective light microscopy (Fig. 2 ▸). Almost all pores were filled with a single, crystal-containing cell in a monolayer, limiting the expected multiple crystal hits by a single XFEL pulse during diffraction data collection.

Sample dehydration after the removal of mother liquor through the pores of a micro-patterned chip has been considered as an obstacle in FT-SFX experiments, and various technical solutions have been designed to keep isolated crystals hydrated during chip loading, transport to the beamline and data collection (Lieske *et al.*, 2019 ▸; Shelby *et al.*, 2020 ▸; Martiel *et al.*, 2019 ▸). Protein crystals formed in a living cell are to a certain degree intrinsically protected from environmental stress if the cell membrane is intact. However, due to the presence of aquaporins in the cellular membrane, strong evaporation pressure on the cell surface will be compensated by water efflux from inside the cell, damaging the contained crystals. To prevent the dehydration of cells and crystals, loading of the crystal-containing cells onto the chip surface was performed in a humidified environment. The ‘humidor’, a specific humidifying feature of the Roadrunner II chips (Lieske *et al.*, 2019 ▸), was slid over the prepared chips during transport and mounting.

### 3.3. FT-SFX structure determination   

**Figure 3:**
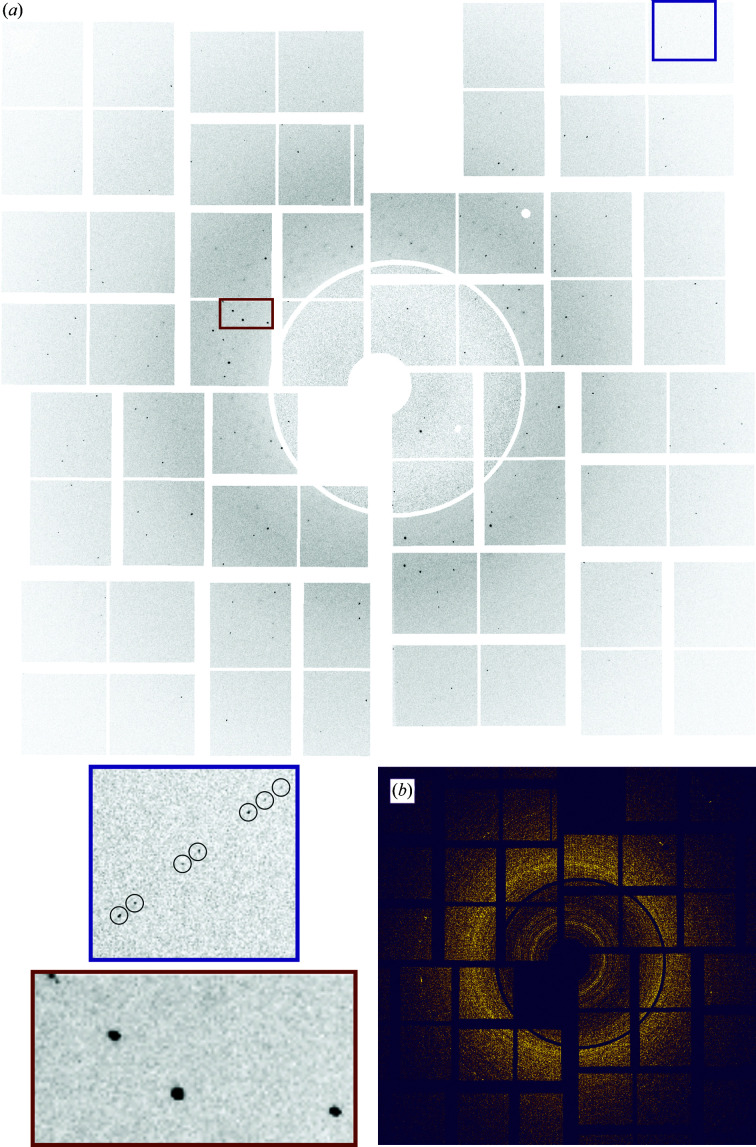
(*a*) An individual diffraction pattern of an intracellular HEX-1 crystal in a Sf9 cell, recorded at room temperature on a Roadrunner II chip with a CSPAD detector. The maximum resolution was limited by the setup to 2.0 Å at the detector edges. Higher resolution peaks of up to 1.70 Å could be detected in the detector corners (blue inset). The shape of the peaks indicates low mosaicity (red inset). (*b*) Virtual powder diffraction pattern of HEX-1 crystals recorded *in cellulo* shows a homogeneous distribution of crystal orientations. The sum of detected Bragg peaks detected by *indexamajig* from the *CrystFEL* suite in all images resulting from the data collection from chips 1 and 2 is depicted.

In the humidified helium environment at the MFX end station, detector images from a total of 87 450 X-ray pulses were recorded by the CSPAD detector from raster-scanning chip 1 (43 890 frames) and chip 2 (43 560 frames) within 25 min. Chips 1 and 2 resulted in 13 247 and 6429 frames containing detectable Bragg spots, respectively, yielding hit rates of approximately 30% (chip 1) and 15% (chip 2). Since the chip pores are specifically targeted with the XFEL beam, almost full pore coverage with cells that form HEX-1 crystals with 85% efficiency should result in higher hit rates. However, this is limited by diffraction from empty holes, holes with empty cells, holes with intracellular crystals positioned outside the pore centre and holes with multiple crystals grown either in a single cell or in multiple cells. The recorded diffraction patterns showed an average maximum resolution of 2.3 Å (Supplementary Fig. S1). Individual patterns contained diffraction even at the corners of the detector (Fig. 3 ▸), corresponding to a maximum per-frame resolution of 1.70 Å. This clearly indicates a higher diffraction capacity of the intracellular HEX-1 crystals within the cellular environment than that recorded using the available experimental setup. However, this FT-SFX experiment was part of a protein crystal screening beamtime that immediately followed another beamtime using the Roadrunner II goniometer setup at the MFX end station. Due to the short time for switching between these two shifts there was no time to optimize the goniometer–detector geometry specifically for this experiment, which somewhat limited the achievable resolution. No indications of crystal damage during diffraction data collection were obtained. Calculating the maximum and average per-pattern resolution for each quarter of a split data set resulted in comparable distributions (Supplementary Fig. S1). Only low background contribution was observed in the majority of the diffraction patterns, as exemplarily shown in Fig. 3 ▸(*a*), confirming the minor diffuse scattering from the soft matter of the insect cell embedding the HEX-1 crystals. The efficient removal of cell-culture medium during sample loading was revealed by the absence of significant water scattering in most patterns, which is typically detected around 3.1 Å resolution in room-temperature liquid-jet SFX experiments (Chapman *et al.*, 2011 ▸; Boutet *et al.*, 2012 ▸). Flow alignment of rod-shaped crystals represents another problem that frequently occurs during serial data collection of rod-shaped crystals, if injected into the XFEL beam by a liquid jet. This was previously observed for *in cellulo* grown, but isolated TbCatB crystals (Koopmann *et al.*, 2012 ▸) and for human aquaporin 2 (hAQP2) crystals (Lieske *et al.*, 2019 ▸. The preferred crystal orientation requires an increased number of hits to record a complete diffraction data set, and thus more beamtime. In FT-SFX a similar problem occurs when non-isometric, such as plate-like or needle-shaped, crystals attach in a preferred orientation to the chip surface (Lieske *et al.*, 2019 ▸). Apparently, in the present case the roundish cells prevented such a preferred orientation of the elongated HEX-1 crystals during chip loading, as revealed by a virtual powder pattern exhibiting a uniform orientation distribution of the HEX-1 crystals in the intact cells loaded onto the silicon chip surface [Fig. 3 ▸(*b*)].

Applying the parameters of a primitive hexagonal unit cell, 15 224 of the totally recorded diffraction patterns were successfully indexed (77% indexing rate), including 15% successfully indexed multi-hits from either two or three crystals. Initially, two populations were found, differing in the length of the *c* axis by 0.93 Å (Supplementary Fig. S3). Following indexing with *MOSFLM*, *ASDF*, *XDS* and *stream-grep*, 12 180 patterns originating from a single crystal population with unit-cell parameters *a* = *b* = 58.73 ± 0.08, *c* = 192.83 ± 0.24 Å, α = β = 90 ± 0.06°, γ = 120 ± 0.07° were combined. Within the error of the respective measurements, the *c* axis of the observed unit cell is slightly shortened compared with that of the HEX-1 reference structure, while the *a* and *b* axes are slightly elongated (*a* = *b* = 57.43, *c* = 196.98 Å; PDB entry 1khi; Yuan *et al.*, 2003 ▸). The refined unit-cell parameters extracted from X-ray powder diffraction tests of HEX-1 crystal-containing insect cells on a synchrotron beamline (*a* = *b* = 58.01, *c* = 195.27 Å; Lahey-Rudolph *et al.*, 2020 ▸) had already indicated a similar composition of the *in vitro* and *in cellulo* grown HEX-1 crystals, both belonging to space group *P*6_5_22 and containing one HEX-1 molecule in the asymmetric unit. Thus, the unit-cell shortening of *c*, together with a slight expansion of *a* and *b*, are more likely to be attributable to an impact of the different buffers in which the cells were floating (ESF 921 insect-cell culture medium versus Tris-buffered saline) during diffraction data collection than to an effect of the intracellular environment of the living insect cells during crystal growth.

It was possible to assemble a complete high-quality data set to elucidate the FT-SFX structure of HEX-1 using only diffraction data obtained from chip 1, collected within 12 min. 8579 indexed crystal diffraction snapshots filtered using the *stream-grep* script were merged into the resulting stream file with *indexamajig*. Molecular replacement using the coordinates of the *N. crassa* HEX-1 reference structure (PDB entry 1khi) as a search model and subsequent refinement resulted in a structural model of *in cellulo* crystallized HEX-1 at 1.80 Å resolution. The resolution is comparable with that obtained by synchrotron diffraction of a single native 0.2 × 0.2 × 0.2 mm HEX-1 crystal at 100 K (1.8 Å; Yuan *et al.*, 2003 ▸). However, considering the previously mentioned limits imposed by the goniometer–detector geometry on the maximum recordable resolution in this study, a better diffraction capability of the HEX-1 *in cellulo* crystals cannot be excluded. As expected, increasing the number of indexed and *stream-grep* filtered patterns to 12 180 (from 19 676 recorded hits) by combination of diffraction data from chips 1 and 2 slightly improved the quality parameters of the data set, for example the signal-to-noise ratio (5.17 to 5.80), CC* (0.987 to 0.991) and CC_1/2_ (0.951 to 0.964), as a consequence of the increased multiplicity of the individual Bragg peaks (107.66 to 130.14). The maximum resolution of the data set improved by 0.1 Å to 1.70 Å (Table 1 ▸).

### 3.4. HEX-1 structure   

**Figure 4:**
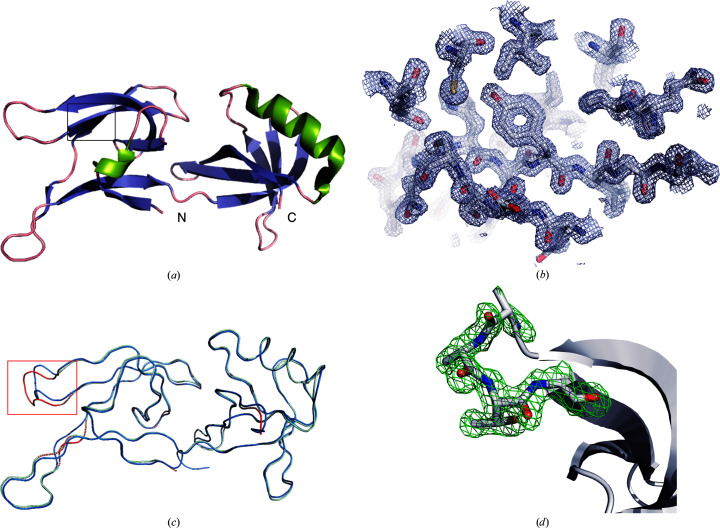
Details of the HEX-1 structure. (*a*) Overall structure of HEX-1 in cartoon representation. (*b*) Representative region of the electron-density map of HEX-1 obtained by FT-SFX of intracellular crystals at 1.8 Å resolution from a single chip (PDB entry 7asx). The detailed region is marked by a black box in (*a*). (*c*) Structural homology of HEX-1 crystallized in insect cells and HEX-1 purified from *E. coli* and crystallized by sitting-drop vapour diffusion. A backbone representation of the HEX-1 structure (green) obtained from *in cellulo* diffraction is superimposed with that of the HEX-1 reference structure (PDB code 1khi; blue). The average r.m.s.d. is 0.47 Å for equivalent C^α^ atoms. The only region showing major structural differences with r.m.s.d.s above 0.6 Å is highlighted by the red box. (*d*) OMIT map of residues 62–65 of the FT-SFX HEX-1 structure with the same residues in stick representation. The *F*_o_ − *F*_c_ map (green) of the region of largest r.m.s.d.s with the reference structure, highlighted in (*c*), is contoured at 3σ.

The 19 kDa protein HEX-1 forms a two-domain structure consisting of mutually perpendicular antiparallel β-barrels. The N-terminal domain is formed by six antiparallel β-strands and a single short helix, while the C-terminal domain consists of a five-stranded β-barrel and two helices [Fig. 4 ▸(*a*)]. The resulting high-quality electron density obtained by FT-SFX provides a high level of detail, as shown for part of the HEX-1 polypeptide chain in Fig. 4 ▸(*b*). No interpretable electron density is observed for the N-terminal (residues 1–30) and C-terminal (residues 171–176) HEX-1 residues and for residue His160, indicating high flexibility of these parts of the structure. Likewise, the side chains of residues Arg41, Gln78, Gln112, Asp113, Asn139, Lys143, Glu146, Ser147, Asp159 and Arg162 are poorly defined or undefined. Two alternate conformations were found for Val157. Comparison of the HEX-1 structures obtained by crystallization and X-ray diffraction in living insect cells with the reference structure revealed no major differences in the overall conformation of the HEX-1 monomer. In general, the structures are similar, with overall root-mean-square deviations (r.m.s.d.s) of 0.466 Å (chip 1 structure) and 0.467 Å (chips 1 and 2 structure) for 141 C^α^ atoms. Small deviations are restricted to the loop regions as well as the linker region connecting the N- and C-terminal domains, which are known to be characterized by increased flexibility [Fig. 4 ▸(*c*)]. To rule out phase bias imposed by phase determination applying the MR approach with the similar reference structure as the search model, an OMIT map was calculated for residues 62–65 located in the most deviating loop, which defines the atomic positions of the HEX-1 *in cellulo* model well [Fig. 4 ▸(*d*)].

Three different patterns of strong intermolecular inter­actions have been described to stabilize the crystal lattice of HEX-1, representing the structural basis of its intrinsic tendency for self-assembly (Yuan *et al.*, 2003 ▸). All residues previously identified to establish these salt bridges and hydrogen bonds are well defined in the FT-SFX electron density, revealing conservation of the lattice interactions in the *in cellulo* grown HEX-1 crystals (Supplementary Table S1, Supplementary Fig. S4). Independent of the environmental conditions in artificial crystallization chambers (Yuan *et al.*, 2003 ▸) or within the cytosol and the nucleus of a living insect cell, HEX-1 forms a polymeric helical spiral with 12 monomers per full turn [Supplementary Fig. S5(*a*)]. Interaction of a spiral with six identical neighbours results in the sixfold symmetry of the crystals that is consistently observed in native Woronin bodies (Markham & Collinge, 1987 ▸) [Supplementary Fig. S5(*b*)]. Consequently, the intrinsic crystallization tendency of HEX-1 appears to be maintained as long as the environmental pH does not prevent the formation of the intermolecular interaction patterns.

## 4. Conclusion   

In this study, we exploited the advantages of FT-SFX to establish a novel approach for high-resolution structure elucidation by *in cellulo* diffraction of micrometre-sized protein crystals within living insect cells. Within 12 min of LCLS beamtime, sufficient diffraction data to elucidate the HEX-1 structure at 1.8 Å resolution were recorded, supported by the sixfold symmetry of the HEX-1 crystals. Pre-positioning of the crystal-containing cells on the silicon chips increased the hit rate by up to 30%, allowing a much more efficient use of the instrument and the sample compared with previous GDVN injection of isolated *in cellulo* grown crystals (Nass *et al.*, 2020 ▸; Redecke *et al.*, 2013 ▸). The direct use of intact, crystal-containing insect cells avoids the time-consuming isolation and enrichment of the crystalline material, restricting the sample-preparation time to a minimum. Problems associated with the usage of intact cells for liquid-jet injection, particularly settling effects of the large cells in the sample reservoir and instable jets due to clogging of GDVN nozzles by the soft matter of the cells, are prevented. Using crystals in intact cells for diffraction protects them from environmental stress during transport, chip loading and diffraction, overcoming some of the challenges reported for the handling of pure protein crystals, for example shearing forces during pipetting (Lieske *et al.*, 2019 ▸), crystal clustering during removal of the surrounding liquid through the chip pores (Soares *et al.*, 2014 ▸) or rapid dehydration after chip loading (Martiel *et al.*, 2019 ▸). The soft matter and the large amounts of water within the living cells irradiated by the XFEL pulse did not impact crystal diffraction. Background levels were sufficiently low to recover diffraction signals to high resolution (1.71 Å), as previously observed (Tsutsui *et al.*, 2015 ▸; Baskaran *et al.*, 2015 ▸; Sawaya *et al.*, 2014 ▸). The reason for the occurrence of two dominating populations of hexagonal unit cells in the recorded diffraction patterns of the HEX-1 *in cellulo* crystals that differ only in the length of the *c* axis needs to be further investigated. However, an impact of the different crystal morphologies that have been observed or the environmental conditions in the cytosol and in the nucleus, where the HEX-1 crystals grow, appears to be reasonable.

Our approach can easily be adopted for other serial diffraction experiments at XFELs for crystalline systems with sufficient diffraction signal at room temperature. The main advantages of fixed-target crystal delivery, lower sample consumption and higher hit rates, have also successfully been exploited for serial diffraction data collection at synchrotron sources, also enabling time-resolved data collection (Roedig *et al.*, 2016 ▸; Meents *et al.*, 2017 ▸; Owen *et al.*, 2017 ▸; Weinert *et al.*, 2017 ▸; Schulz *et al.*, 2018 ▸; Mehrabi *et al.*, 2019 ▸, 2020 ▸). Thus, the fixed-target *in cellulo* diffraction approach presented in this study also provides the possibility of improving SSX experiments in terms of sample consumption and data-collection time. This is important as beamtime at an XFEL is very expensive and difficult to obtain. The application of micro-patterned silicon chips for serial collection of diffraction data from crystals directly in living cells offers huge potential for the straightforward structure elucidation of proteins that form intracellular crystals. This includes soluble proteins in general, even those containing post-translational modifications or in complex with small molecules (Redecke *et al.*, 2013 ▸; Nass *et al.*, 2020 ▸), for which no general limitations have so far been ascertained. Except for the coral fluorescent protein Xpa (Tsutsui *et al.*, 2015 ▸) and HEX-1, all recombinant proteins that produced intracellular crystals were identified by accident and were not predicted to crystallize. In contrast, membrane proteins that need to stay embedded in or attached to their respective membranes are suggested to be less accessible for *in cellulo* crystallization, which needs to be studied in detail in the future. Since the living cell represents an environment that is very close to physiological conditions for most proteins, insights into protein dynamics by time-resolved X-ray crystallography (TRX; Orville, 2020 ▸; Pearson & Mehrabi, 2020 ▸) using intracellular crystals could provide new insights, for example for drug-screening efforts. However, any compounds delivered by photoactivatable caged compounds to induce dynamics need to overcome the cell membrane, representing a significant bottleneck, in addition to other limitations. Protein dynamics directly triggered by light appear to be more accessible if the crystal contacts remain stable during the reaction. Thus, the establishment and validation of TRX using *in cellulo* grown crystals represents a challenging but important task in the future, along with a more detailed investigation of the intracellular crystallization process itself.

## 5. Related literature   

The following reference is cited in the supporting information for this article: Krissinel & Henrick (2007 ▸). 

## Supplementary Material

PDB reference: *Neurospora crassa* HEX-1, 7asi

PDB reference: 7asx

Supplementary Table and Figures. DOI: 10.1107/S2052252521005297/zf5016sup1.pdf

## Figures and Tables

**Table 1:** FT-SFX data-collection and refinement statistics for *N. crassa* HEX-1 loaded onto Roadrunner II chips Values in parentheses are for the highest resolution shell.

	Chip 1	Chips 1 and 2
Data collection		
PDB code	7asx	7asi
No. of frames collected	43840	87450
No. of *Cheetah* hits [hit rate, %]	13247 [30.2]	19676 [22.5]
No. of indexed patterns [indexing rate, %]	11279 [85.1]	15224 [77.4]
No. of merged patterns	8579	12180
Resolution range (Å)	29.37–1.80 (1.86–1.80)	39.90–1.70 (1.744–1.704)
Space group	*P*6_5_22	
*a*, *b*, *c* (Å)	58.75, 58.75, 192.83	
α, β, γ (°)	90, 90, 120	
Mean *I*/σ(*I*)	5.17 (1.42)	5.42 (0.70)
CC_1/2_	0.951 (0.340)	0.964 (0.160)
CC*	0.9873 (0.740)	0.991 (0.525)
*R*_split_ † (%)	17.22 (89.08)	16.24 (189.26)
Completeness (%)	99.95 (100.00)	99.99 (99.86)
Multiplicity	107.66 (29.10)	124.12 (16.04)
Refinement		
Resolution (Å)	2.37–1.80 (1.864–1.800)	39.90–1.704 (1.744–1.704)
Total reflections	2079122	2809415
Unique reflections	19213 (1859)	22635 (1469)
Wilson *B* factor (Å^2^)	17.33	19.11
Reflections used in refinement	19211 (1860)	24698 (1430)
Reflections used for *R*_free_	1541 (149)	1963 (114)
*R*_work_	0.226 (0.362)	0.219 (0.481)
*R*_free_	0.273 (0.366)	0.264 (0.396)
No. of non-H atoms		
Total	1239	1210
Macromolecules	1111	1116
Solvent	128	94
No. of protein residues	141	141
R.m.s.d., bonds (Å)	0.002	0.012
R.m.s.d., angles (°)	0.53	1.316
Ramachandran favoured (%)	97.12	97.20
Ramachandran allowed (%)	2.88	2.82
Ramachandran outliers (%)	0.00	0.00
Rotamer outliers (%)	0.00	0.00
Clashscore	1.34	2.28
Average *B* factor (Å^2^)		
Overall	24.00	25.30
Macromolecules	20.87	21.19
Solvent	36.48	39.09

† *R*_split_ as defined in White *et al.* (2012 ▸).
