# Supplementary material for: Fixed-target serial femtosecond crystallography using in cellulo grown microcrystals
Source: IUCrJ. 2021 Jun 18;8(Pt 4):665–77. doi: 10.1107/S2052252521005297 (PMC8256716; doi:10.1107/S2052252521005297)
Supplement: Supplementary file 1 [file m-08-00665-sup1.pdf]

IUCrJ

Volume 8 (2021)

Supporting information for article:

Fixed-target serial femtosecond crystallography using *in cellulo* grown microcrystals

J. Mia Lahey-Rudolph, Robert Schönherr, Miriam Barthelmess, Pontus Fischer, Carolin Seuring, Armin Wagner, Alke Meents and Lars Redecke

Table S1 Interactions of HEX-1 monomers forming the crystal lattice.

Group1	Monomer 1	Distance	Monomer 2	Type
I	ARG 41[NH1]	3.57	ASP 44[OD1]	Salt bridge
I	ARG 41[NH2]	2.46	ASP 44[OD1]	Salt bridge
I	ARG 41[NH1]	3.04	ASP 44[OD2]	Salt bridge
I	ARG 41[NH2]	3.50	ASP 44[OD2]	Salt bridge
I	HIS 39[NE2]	3.59	ASP 44[OD2]	Salt bridge
I	ASP 44[OD1]	3.57	ARG 41[NH1]	Salt bridge
I	ASP 44[OD1]	2.46	ARG 41[NH2]	Salt bridge
I	ASP 44[OD2]	3.04	ARG 41[NH1]	Salt bridge
I	ASP 44[OD2]	3.50	ARG 41[NH2]	Salt bridge
I	ASP 44[OD2]	3.59	HIS 39[NE2]	Salt bridge
I	ARG 41[HH12]	2.26	ASP 44[OD2]	H bond
I	GLN 105[H]	1.95	SER 92[O]	H bond
I	GLU 121[H]	2.28	SER 92[OG]	H bond
II	ARG 68[NE]	3.61	GLU 81[OE1]	Salt bridge
II	ARG 68[NH2]	2.87	GLU 81[OE1]	Salt bridge
II	ARG 68[NE]	2.61	GLU 81[OE2]	Salt bridge
II	ARG 68[NH2]	3.47	GLU 81[OE2]	Salt bridge
II	GLU 81[OE1]	3.61	ARG 68[NE]	Salt bridge
II	GLU 81[OE1]	2.87	ARG 68[NH2]	Salt bridge
II	GLU 81[OE2]	2.61	ARG 68[NE]	Salt bridge
II	GLU 81[OE2]	3.47	ARG 68[NH2]	Salt bridge
II	SER 84[OG]	2.92	GLN 49[OE1]	H bond
II	GLN 49[OE1]	2.92	SER 84[OG]	H bond
III	ILE 56[N]	2.56	GLN 127[OE1]	H bond
III	GLN 134[HE22]	2.28	VAL 125[O]	H bond

Interactions were identified using the PDBePISA server (Krissinel & Henrick, 2007 www.ebi.ac.uk/pdbe/pisa/);
[1] as defined by (Yuan *et al.*, 2003).

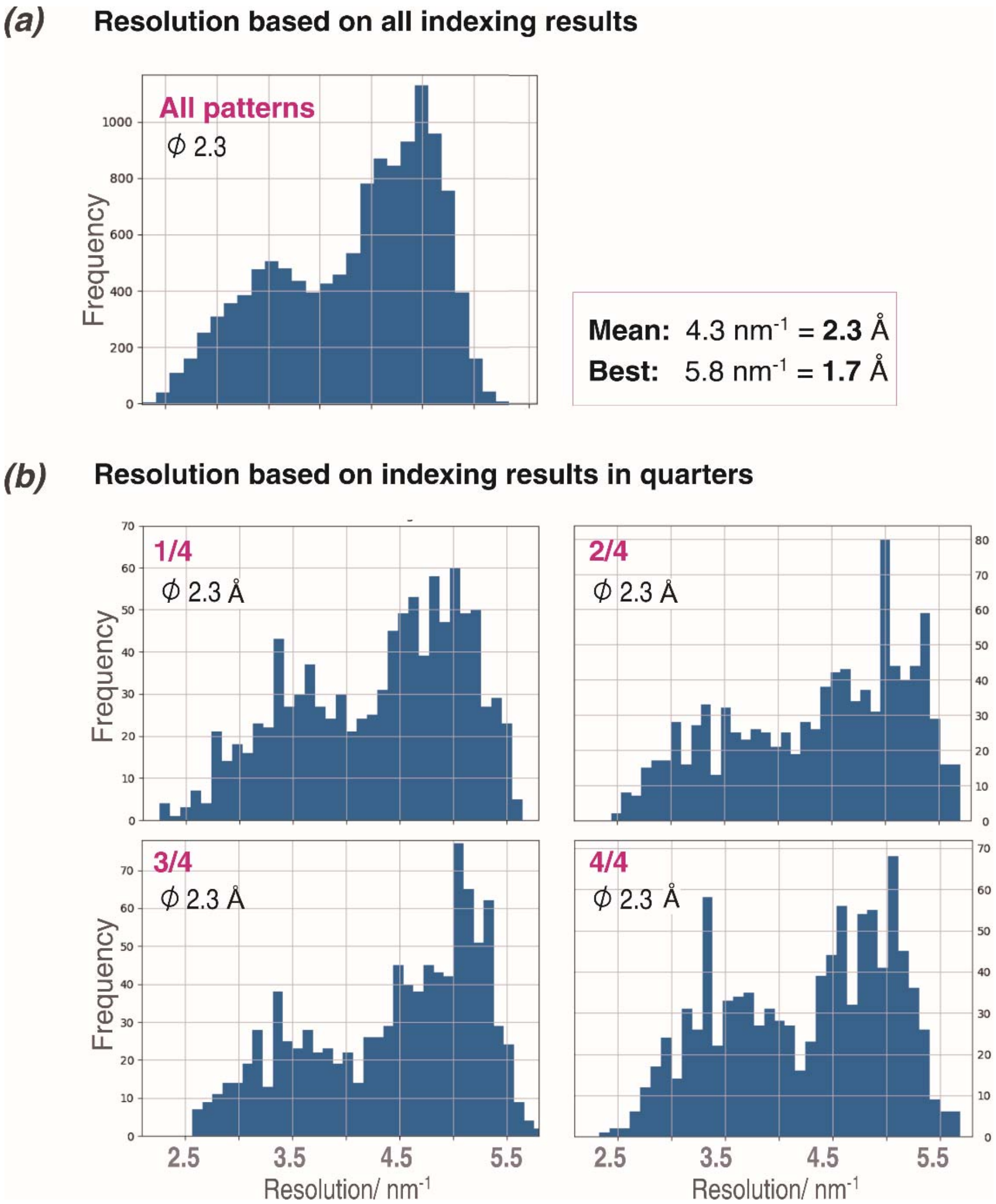

Figure S1 Histograms of the average resolution based on indexing results as determined by CrystFEL. Resolution in nm^{-1} is shown on the x-axis, frequency of patterns with detected Bragg-peaks to this resolution-shell are presented on the y-axis. The distribution of resolution based on all indexing results were extracted from *(a)* the final streamfile including patterns from chip 1 and chip 2 after stream-grep. *(b)* Then, the streamfile with indexing results from diffraction patterns collected with chip 1 was ordered according to the sequence of data collection and sequentially cut in quarters with truncate-stream of the CrystFEL suite, mean and best resolutions were determined with ave-resolution as before. The average resolution of 2.3 Å and the best resolution of 1.7 Å remain the same throughout the quarters of diffraction data collection.

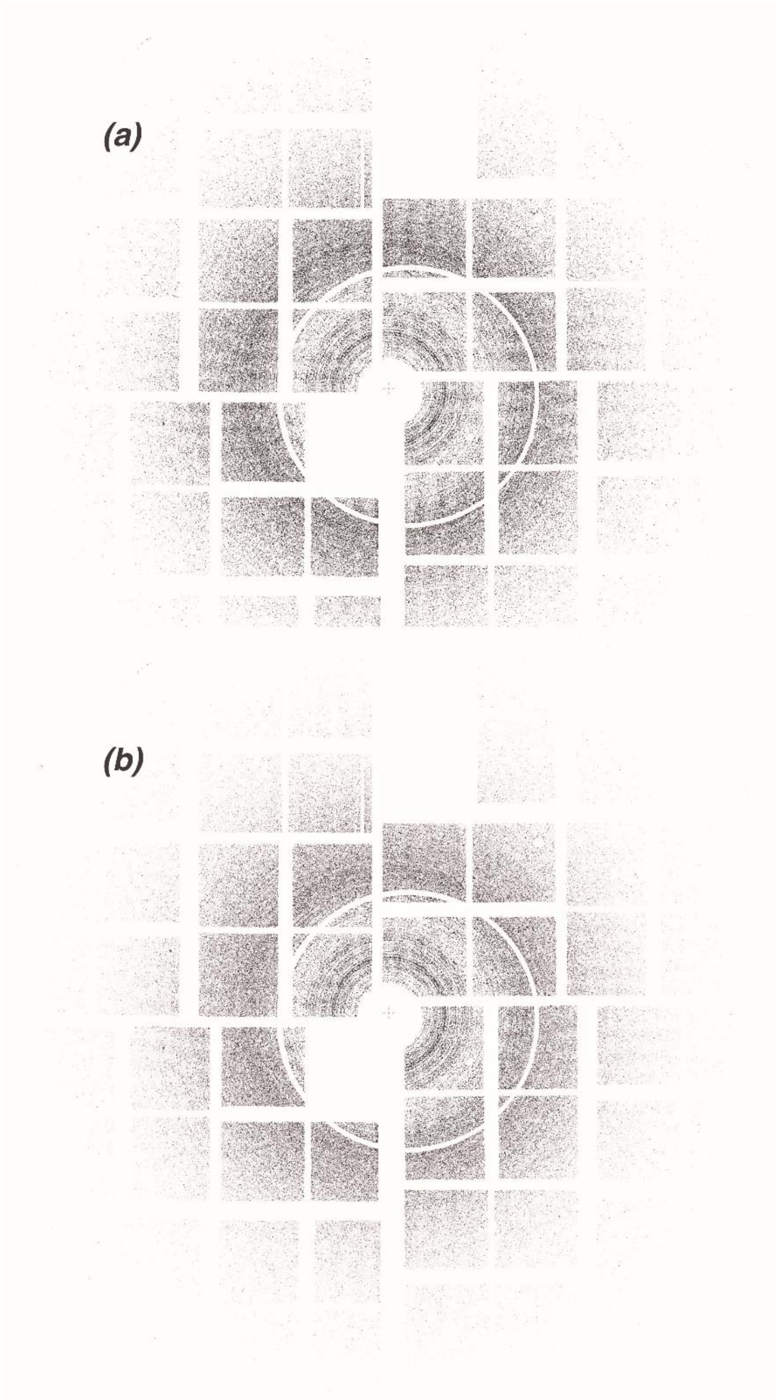

Figure S2 Virtual powder pattern of FT-SFX HEX-1 *in-cellulo* crystal diffraction does not show a preferred orientation of the crystals. Depicted is the sum of all Bragg peaks found by *indexamajig* of the CrystFEL suite, *(a)* of indexed diffraction patterns resulting from measuring one chip, as opposed to *(b)* of indexed diffraction patterns resulting from the measurements on two chips. CSPAD panels and the non-centrosymmetric shadow of the goniometer setup are masked.

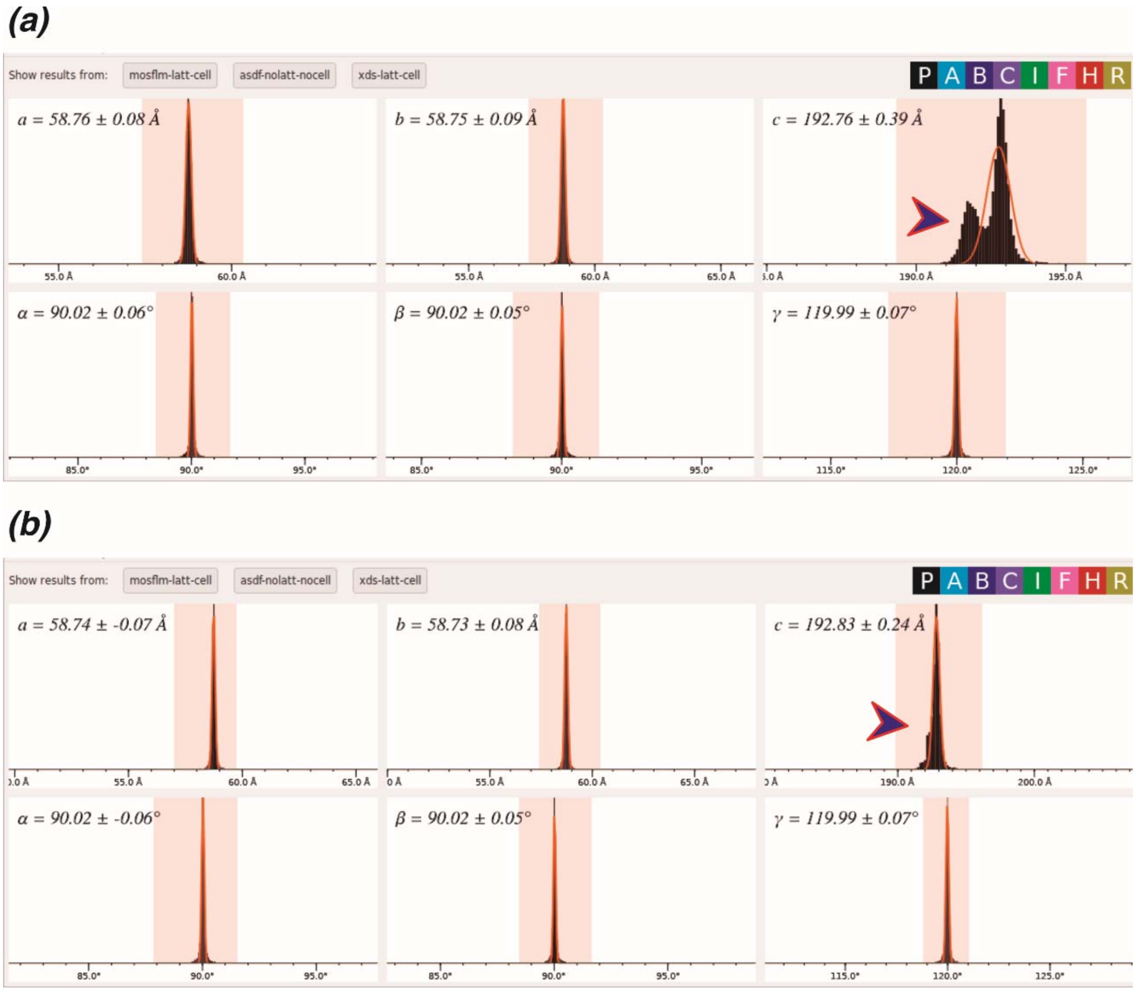

Figure S3 Cell parameter histogram of HEX-1 before *(a)* and after *(b)* stream-grep. Indexing algorithms mosflm, adsf and XDS were used, allowing the detection of multiple lattices in one pattern. Two distinct populations are visible with cell-parameter c of 192.76 Å and a smaller one c = 191.83 Å, highlighted with arrows. The graphics were made with the *cell_explorer* script of the CrystFEL suite.

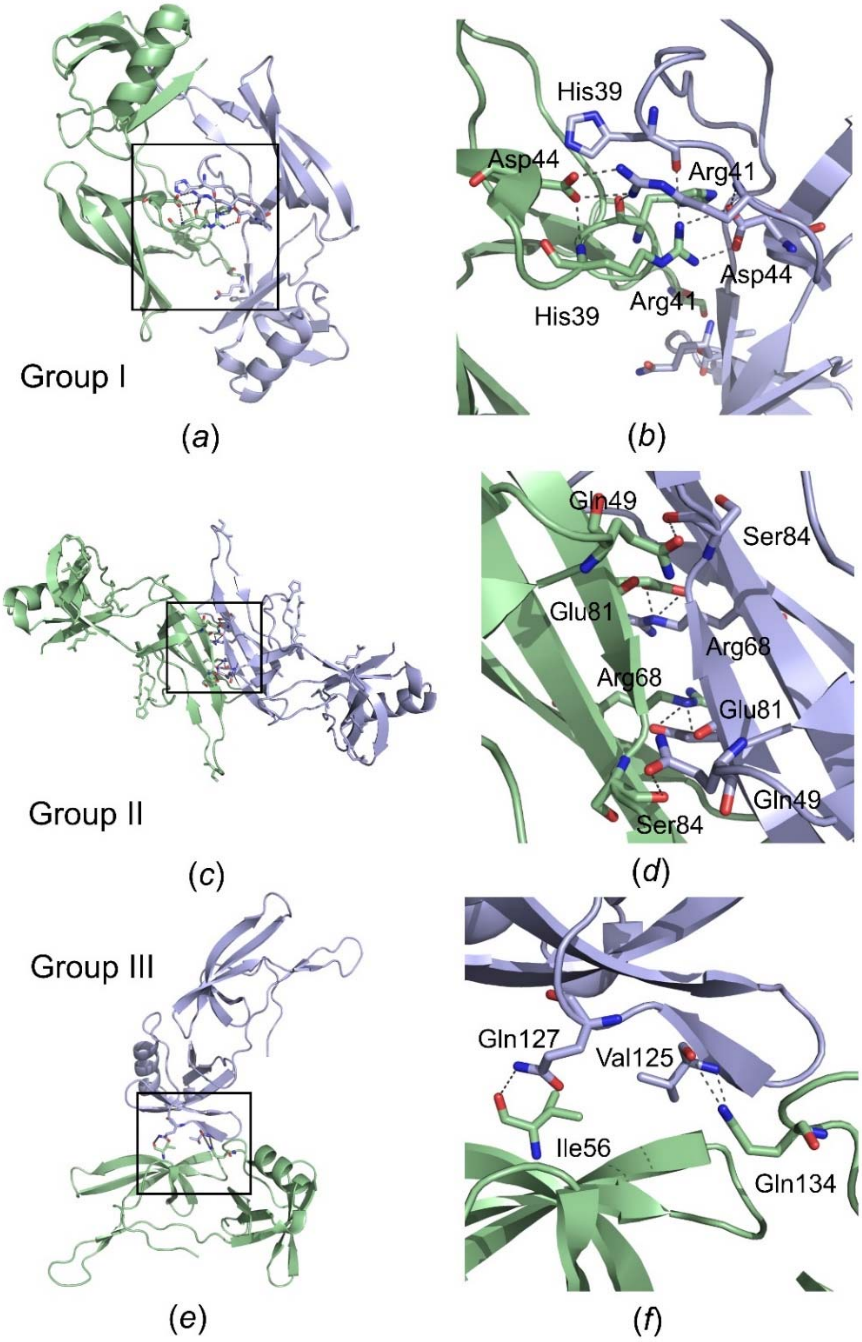

Figure S4 Intermolecular interactions between HEX-1 molecules. Overall views of interacting molecules in blue and green are shown in the left panels and magnified views in the right panels. (*a, b*) Group I interactions include the central N-terminal salt bridges between Arg41 and Asp44 stabilized by His39 and N- to C-terminal domain contacts. (*c, d*) Group II interactions link the N-terminal domains by salt bridges between Arg68 and Glu81 as well as flanking hydrogen bonds formed by Ser84/Gln49 and Ser61/Glu81. (*e, f*) Interactions of the C-terminal amino acids Gln127 and Val125 of one molecule with the N-terminal Ile56 and C-terminal Gln134 of the other molecule are classified as group III interactions. Definitions of the interaction classes as previously described in Yuan et al.(2003).

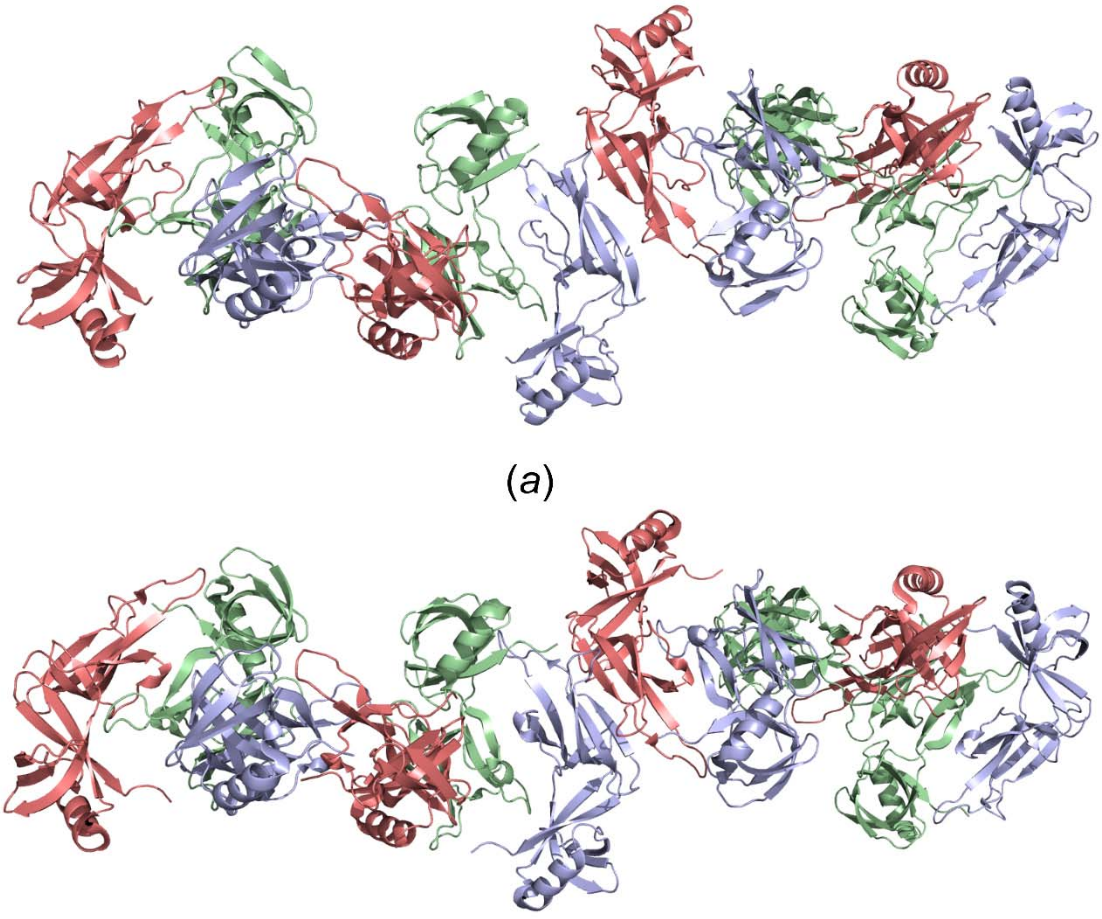

(*a*)

(*b*)

Figure S5 Molecular assembly of HEX-1 forming the crystal lattice. (*a*) A coiled filament characterized by 12 alternating HEX-1 molecules per turn (shown in blue, green, and red) is formed by group I and group II interactions, almost superimposable to that previously described by Yuan *et al.* (2003; PDB 1KHI) (*b*).
